# Organized adrenal hematoma mimicking an adrenocortical carcinoma

**DOI:** 10.1210/jcemcr/luag206

**Published:** 2026-07-27

**Authors:** Takahiro Asano, Yasushi Oiwa, Mitsuhiro Kometani, Takashi Yoneda

**Affiliations:** Department of Internal Medicine, Houju Memorial Hospital, Nomi, Ishikawa 923-1226, Japan; Department of Health Promotion and Medicine of Future, Kanazawa University Graduate School of Medicine, Kanazawa, Ishikawa 920-8641, Japan; Department of Internal Medicine, Houju Memorial Hospital, Nomi, Ishikawa 923-1226, Japan; Department of Health Promotion and Medicine of Future, Kanazawa University Graduate School of Medicine, Kanazawa, Ishikawa 920-8641, Japan; Next-Generation Hypertension Strategy Center, Kanazawa University Hospital, Kanazawa, Ishikawa 920-8641, Japan; Department of Internal Medicine, Houju Memorial Hospital, Nomi, Ishikawa 923-1226, Japan; Department of Health Promotion and Medicine of Future, Kanazawa University Graduate School of Medicine, Kanazawa, Ishikawa 920-8641, Japan; Next-Generation Hypertension Strategy Center, Kanazawa University Hospital, Kanazawa, Ishikawa 920-8641, Japan

**Keywords:** adrenal hemorrhage, adrenal hematoma, anticoagulant

## Image legend

A 79-year-old man receiving edoxaban for prevention of recurrent deep vein thrombosis, with a history of recurrent falls, was found to have a left adrenal mass after a fall. Panel A shows a 56-mm heterogeneous lesion measuring 40 Hounsfield units on non-contrast computed tomography. Panel B, obtained about 3 weeks later, shows no internal enhancement on contrast-enhanced computed tomography. Panel C shows a lipid-poor lesion on magnetic resonance imaging. The mass remained stable for 5 months and then enlarged to 73 mm over 6 months and to 83 mm over the next 4 months (Panel D). Panel E shows mild fluorodeoxyglucose uptake in the adrenal lesion (maximum standardized uptake value, 2.1) and similar uptake in a right thigh lesion, raising concern for metastatic adrenocortical carcinoma. Hormonal workup and iodine-123 metaiodobenzylguanidine scintigraphy were negative. Laparoscopic adrenalectomy showed organized hematoma with calcification and hemosiderin deposition (Panel F, low-power view; scale bar = 10 mm); the thigh lesion biopsy showed the same pathology. Anticoagulant use and falls-related trauma were possible contributors to adrenal hemorrhage. Organized hematoma should be considered in enlarging adrenal masses because metabolic imaging can falsely suggest malignancy [1]. Progressive enlargement also supported surgery under current guidance for suspicious adrenal masses [2].

**Figure:**
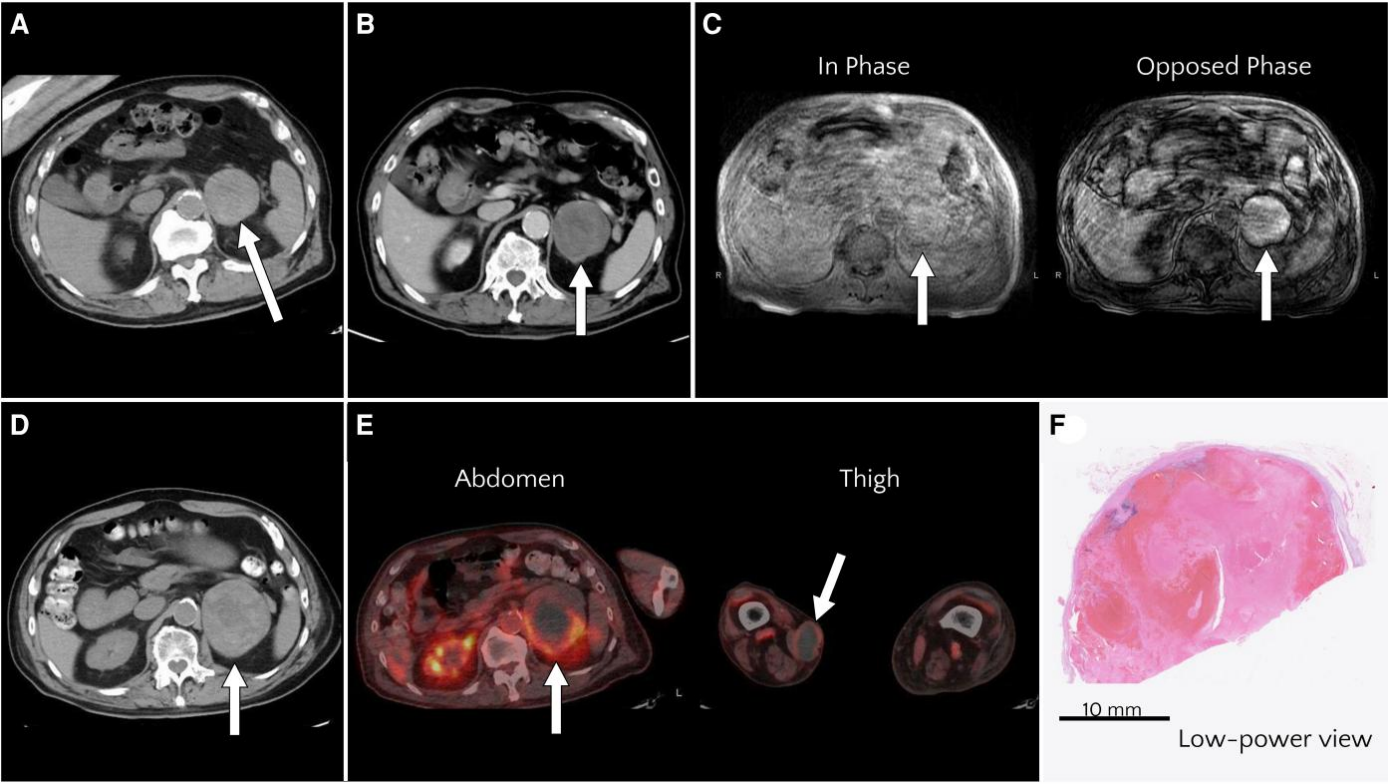
Imaging and histopathological findings of an enlarging left adrenal mass

## Contributors

All authors made substantial contributions to the work. T.A., M.K., and T.Y. were involved in the diagnosis, clinical management, and treatment decision-making for the patient. T.A., Y.O., and M.K. contributed to the conception of the report and drafted the manuscript. T.A., Y.O., and M.K. also critically revised the manuscript for important intellectual content. T.Y. critically reviewed the manuscript and provided important intellectual input. All authors reviewed and approved the final version of the manuscript and agree to be accountable for all aspects of the work.
